# Awareness of hypertension and its impact on blood pressure control among elderly nigerians: report from the Ibadan study of aging

**DOI:** 10.11604/pamj.2017.27.190.11682

**Published:** 2017-07-13

**Authors:** Yemi Raheem Raji, Taiwo Abiona, Oye Gureje

**Affiliations:** 1 Department of Medicine, College of Medicine, University of Ibadan, Ibadan, Nigeria; 2 Department of Community Medicine, College of Medicine, University of Ibadan, Ibadan, Nigeria; 3 Department of Psychiatry, College of Medicine, University of Ibadan, Ibadan, Nigeria

**Keywords:** Awareness, elderly, hypertension, Nigeria

## Abstract

**Introduction:**

Hypertension is highly prevalent among the elderly. Its awareness has a direct influence on control through drug adherence. Earlier studies have shown that awareness of hypertension is low among sub-Saharan African populations but only a few studies have looked at the prevalence and awareness of hypertension among the elderly.

**Methods:**

The Ibadan Study of Ageing is a longitudinal cohort study of the mental and physical health status as well as the functioning of elderly persons residing in the Yoruba-speaking areas of Nigeria. Study was conducted in multiple waves from 2003/2004 to 2009. This report is based on the sample studied in 2007 (N = 1469). Respondents, aged ≥ 65 years, were assessed for the presence of hypertension, its awareness, receipt of and adherence to medication for the condition, and effectiveness of treatment on the control of blood pressure. Blood pressure was measured with the use of digital monitors (Omron MS - 2 Basic Model). Awareness of the diagnosis of hypertension was ascertained by self-reports. We explored social, economic, demographic and clinical correlates of the presence of hypertension, its awareness and control using multiple logistic regression analyses.

**Results:**

The sample was composed of 809 (55.1%) females and 666 (44.9%) males. The mean age of the participants was 76.9 ± 8.4 years. Hypertension (defined as previous diagnosis by a health provider or a measured blood pressure higher than or equal to 140/90 mm Hg) was recorded in 973 (62.2%) participants, with females having a prevalence of 61.4% and males that of 70.1%. Other than female gender, residing in urban/semi urban areas and being overweight or obesity were associated with the occurrence of hypertension. Among those assessed to have hypertension, 78% were not previously aware of its presence. Factors independently associated with lack of awareness of hypertension included low socioeconomic class (OR 8.21, 95% CI 3.72-18.11, P < 0.001), and BMI >25kg/m^2^ (OR 3.11, 95% CI 1.36-7.09, P < 0.009). Among those who were aware of the presence of hypertension and were on treatment, 77.3% still had uncontrolled hypertension. Only obesity or overweight (OR 5.56, 95% CI 1.35 - 22.83, P < 0.016) was independently associated with poor blood pressure control.

**Conclusion:**

The prevalence of hypertension among elderly Nigerians is high and those affected are often not aware of having the condition. Only a minority of those who receive treatment for the condition have adequate blood pressure control. The findings highlight the need for improved healthcare for the growing population of elderly persons, with particular attention to early detection and effective control of the condition.

## Introduction

Systemic hypertension is a disease of public health importance. As the single most important risk factor for cardiovascular death and disability, it accounted for 7.5 million and 9.4 million deaths worldwide in 2004 and 2010 respectively [1]. In the year 2000, about 26.4% of the world adult population were living with hypertension and it is projected that this figure will increase to about 29.2% by 2025 [2]. Low and middle-income countries (LMIC) bear the majority of the burden associated with hypertension. Estimated total number of adults with hypertension in high income countries in 2000 was 333 Million while it was 654 Million in LMIC [3]. With an expected 60% increase by 2025, it is estimated that the total number of affected persons will rise to 1.56 Billion adults globally [3, 4]. Among the sequelae of uncontrolled hypertension are stroke, multi infarct dementia, heart failure, myocardial infarction and renal failure [5]. The burden of hypertension is more among the elderly population in view of its higher prevalence and associated morbidity and mortality in this age group [6]. Age-dependent positive association exists between systolic blood pressure and diastolic blood pressure (SBP/DBP) on the one hand and stroke and ischaemic heart disease on the other hand [7]. Increasing age is associated with a progressive rise in risk of vascular mortality with a 20 mmHg rise in SBP above 125 mmHg or 10 mmHg above DBP of 75 mmHg, and this observed risk is common among the elderly population [6, 8].

Despite the high burden of hypertension, most affected persons are not aware of its presence, thus increasing the occurrence of associated complications, particularly among elderly populations [9, 10]. Awareness of the diagnosis of hypertension is an important determinant of treatment and medication adherence. Awareness of hypertension is high in developed countries compared to developing nations. For example, in the third National Health And Nutrition Examination Survey (NHANES III), awareness of hypertension approached 73% among the United States adult population while in Nigeria only about 30% of persons with the condition was aware of it at the time of diagnosis [1, 11]. Optimal control of hypertension has been shown to reduce the risk of cardiovascular complications, particularly that of SBP which is more prevalent among the elderly population [5, 12]. The knowledge and awareness of the diagnosis as well as of the risk associated with uncontrolled hypertension tend to enhance patients’ adherence to lifestyle modifications and to medications [13, 14]. With a growing elderly population in sub-Saharan Africa, adequate knowledge about the awareness, control and treatment of hypertension will be required to guide the development of policies designed to reduce the burden of hypertension in the population. We carried out a secondary analysis of data from the Ibadan Study of Ageing with the aim of determining the prevalence, awareness, treatment and control of hypertension among the study population.

## Methods

### Sampling

The Ibadan Study of Ageing is a longitudinal community based cohort study of the mental and physical health status as well as the functioning of elderly persons (aged ≥ 60 years) residing in the Yoruba-speaking areas of Nigeria, which consists of eight contiguous states in the Southwestern and North central regions (Lagos, Ogun, Osun, Oyo, Ondo, Ekiti, Kogi and Kwara). The population of these states at the time of study was approximately 25 million people, which was about 22% of the Nigerian population. The baseline characteristics and methodology have been fully described in earlier reports [15, 16], thus only a brief summary is reported here. Respondents were selected using a multistage stratified area probability sampling of households. In households with more than one eligible person available (aged ≥ 60 years and fluent in Yoruba, the language of the study), the Kish table was used to select one respondent. All the eligible respondents were approached after ethical approval was sought and obtained from the institutional review board. A total of 2,152 respondents were recruited for the study with a response of 79% while 3 respondent records were excluded for incomplete data. This baseline assessment was carried out between August 2003 and November 2004. The non-response rate was due to change in address or not found at home after repeated visits rather than refusal. The cohort was followed up in 2007 in the first of 3-wave annual assessments. Of the baseline cohort of 2,149,269 had died and 472 could not be found or refused to take part during 1^st^ follow-up wave. The surviving participants who consented to participate in the 1st wave were 1408. This sample was enlarged in 2007 by the selection and assessment of additional 506 persons from the initial household listings. The new total of 1914 respondents constituted the sample for the 2007 assessments. Of these, 287 respondents with incomplete data and 158 respondents who were less than 65 years of age were excluded from the analysis. The current report is therefore based on 1469 persons who were assessed in 2007.

### Data collection

Assessment was carried out on the participants through face-to face interviews. The interviews were conducted by 24 trained interviewers all of whom had at least a high school education. Many of the interviewers had previously been involved in field surveys and had experience of face-to-face interviews. Interviewers undertook two weeks of training, consisting of an initial 6-day training delivered by one of the authors (OG) (which included item-by-item description of questionnaires, measurement of blood pressure, role plays, as well as other assessments), followed by a further two days of debriefing and review after every interviewer had done two practice assessments in the field. Six supervisors, all of whom were university graduates and had survey experience, underwent the same level of training and monitored the day-to-day implementation of the survey.

### Measures

Along with several other assessments, a checklist of chronic physical and pain conditions was included in the Ibadan Study of Ageing [16]. At the 2007 follow-up, respondents were asked if they had been told by a physician that they had diabetes mellitus or hypertension. Questions were asked about the use of antihypertensive medications and adherence, the presence or past history of complications of hypertension. Also included were self-reported histories of other chronic illnesses. Blood pressure was measured using a validated digital monitor (Omron MS - 2 Basic Model). The measurements were taken in sitting position after at least 5 minutes rest. Three measurements were recorded approximately 5 minutes apart and the average determined. Hypertension was defined using World Health Organization (WHO) definition which defined hypertension as previous diagnosis of hypertension by a physician, use of antihypertensive medications or systolic blood pressure (SBP) of 140 mmHg and above or diastolic blood pressure (DBP) of 90 mmHg or greater (18). Hypertension was adjudged to be controlled if SBP was less than 140 mmHg and DBP was less than 90mmHg in those who reported having been previously told that they had hypertension and were on medication [17]. Diabetes mellitus was based on self-reported previous diagnosis of the condition or use of antidiabetic agents. Transient Ischaemic Attack (TIA) was defined as occurrence of sudden onset of focal or global neurological deficit that resolved within 24 hours of onset [18]. Depression was assessed using the World Health Organization (WHO) Composite International Diagnostic Interview, version 3 (CIDI.3), a fully structured diagnostic interview [19]. As previously described, diagnosis of dementia was made using validated protocols previously used in our setting [20].

### Data analysis

Data was analyzed with the STATA statistical package, version 14. Weights were derived and applied to adjust for the clustering associated with the stratified multistage sampling method. Post stratification to the target age and gender was carried out to adjust for the differences in the sample and the total Nigerian population of persons aged 65 years and over. Continuous variables are expressed as means and standard deviations while categorical variables are expressed as proportions and percentages. Univariate analyses were conducted to determine demographic and clinical variables associated with the presence of hypertension, its awareness by respondents and its control by medication. Multivariate analyses were then employed to identify factors that were independently associated with hypertension, its awareness and blood pressure control. We classified the economic status of participants using an inventory of 21 household and personal items such as chairs, radio, television sets, cookers, and iron (wealth index). Each respondent’s status was determined relative to the median number of possessions in the total sample. Thus, economic status was then classified as low (≤ 0.5 of the median); low-average (> 0.5-1.0); high-average (> 1.0-2.0) or high (> 2).

## Results

The records of one thousand four hundred and sixty nine elderly persons with complete data were analyzed for the study. Of these, 809 (55.1%) were females while 660 (45.9 %) were males. The mean age of the total study population was 76.9±8.4 years. Subjects in the low economic group were 334 (22,7%), average socioeconomic group (low-average and high-average) were 1,017(69.2%) while the subjects in high economic group 118 (8.0%). Five hundred and fifty subjects (37.4%) lived in urban areas, 508 (34.6%) lived in semi-urban while 411 (28.0%) subjects lived in the rural communities (Table 1). Self-reported diabetes mellitus was documented in 64 (4.4%) participants, 64 (4.4%) also reported previous history of transient ischaemic attack (TIA), 121 (8.2%) had dementia, life time depression was reported in 684 (46.6%), depression in the last 12 months was reported in 129 participants (8.8%) (Table 1).

**Table 1:** Demographic and clinical characteristics of all subjects

Parameters	Total N = 1469	Hypertensive N = 973	Normotensive N = 496	P-Value
**Gender**				0.003*
Female	809 (55.1%)	572(58.8%)	237(47.8%)	
Male	666 (44.9%)	409(41.3%)	259 (52.2%)	
**Age Group (years)**				0.206
65 - 69	308 (21.0%)	204(23.6%)	104 (20.9%)	
70 - 74	405 (27.5%)	279(28.6%)	129 (26.1%)	
75 - 79	276 (18.8%)	166(17.0%)	110 (22.2%)	
80+	480 (32.7%)	327(33.5%)	153 (30.8%)	
**Marital status**				0.001*
Married	818 (55.7%)	503(51.7%)	315 (63.5%)	
Not married	651 (44.3%)	470(48.3%)	181 (36.5S.5%)	
Educational level				0.201
No formal education	658 (22.8%)	442 (22.7%)	216 (22.7%)	
Below secondary level	725 (69.4%)	490 (69.1%)	235 (70.2%)	
Above secondary level	86 (7.8%)	65 (8.2.1%)	18 (7.1%)	
**Economic Group**				0.759
< 3	334 (22.8%)	221(22.7%)	113(22.8%)	
3 -5	572 (38.9%)	381 (39.2%)	191 (38.5%)	
6 - 10	445 (30.3%)	291 (29.9%)	154 (31.0%)	
> 10	118 (8.0%)	80(8.2%)	38 (7.7%)	
**Site of study**				0.031*
Urban	550 (37.4%)	385(39.6%)	165 (33.2%)	
Semi Urban	508 (34.6%)	334 (34.3%)	174 (35.1%)	
Rural	411 (28.0%)	254 (25.9%)	157 (31.7%)	
**Ever smoked cigarette**				0.032*
Yes	606 (41.3%)	381 (39.9%)	225 (45.4%)	
No	863 (58.7%)	592 (60.1%)	271 (54.6%)	
**Ever drank alcohol**				0.002*
Yes	612 (41.7%)	378 (38.9%)	234 (47.2%)	
No	857 (58.3%)	595 (61.1%)	262 (52.8%)	
Body Mass Index (kg/m^2^)				0.005*
< 18.5	254 (18.2%)	141 (14.5%)	113 (22.8%)	
18.5 - 24.9	828 (59.3%)	538 (55.3%)	290 (58.5%)	
25 - 29.9	241 (17.3%)	180 (18.4%)	61 (21.2%)	
> 30	72 (5.2%)	61 (6.3%)	11 (2.2%)	
**Diabetes mellitus**				0.630
Present	64 (4.4%)	43 (4.5%)	21 (4.3%)	
Absent	1,405 (95.6%)	930 (95.5%)	475(95.7%)	
**Transient ischaemic attack**				0.852
Previous TIA	64 (4.4%)	46 (4.0%)	18 (3.6%)	
No TIA	1405 (95.6%)	927 (96.0%)	474 5(95.7%)	
Dementia				0.302
Present	121 (8.2%)	83 (8.5%)	38 (7.7%)	
Absent	1,348 (91.8%)	890 (91.5%)	458 (92.3%)
**Lifetime depression**				0.601
Present	684 (46.6%)	473 (48.6%)	211 (42.5%)	
Absent	785 (53.4%)	500 (51.4%)	285 (57.5%)	

TIA - Transient Ischaemic Attack

* Statistically significant

Among the participants, 973 subjects (62.2%) reported history of hypertension or were found to years and female accounted for 620 (62.1%). Other characteristics are shown in Table 1. On univariate analysis, occurrence of hypertension was associated with female gender, not currently being married (mostly being widowed), residing in urban or semi urban areas and being overweight or obese (Table 1). In multivariate analysis only being overweight or obese (OR 3.78, 1.47-9.40, P < 0.006) and living in urban/semi urban areas (OR 1.38, 95% CI 1.02-1.88, P < 0.039) were independently associated with hypertension, while male gender was less likely to be associated with hypertension (OR 0.62, 95% CI 0.48-0.79, P < 0.001) (Table 2). Seven hundred and fifty nine participants (78%) with high blood pressure were not aware of having hypertension. On univariate analysis, individuals who were not aware of their hypertension were more likely to be older than 69 years, currently unmarried, of low socioeconomic class, overweight or obese (BMI > 25kg/m^2^) and were less likely to report having diabetes mellitus and dementia (Table 3).

**Table 2:** Factors independently associated with hypertension among the elderly

Factors	Odd Ratio	95% CI	P-value
Female gender	0.62	0.4814 - 0.7921	0.001^*^
Age > 69 years	1.16	0.7815 - 1.7242	0.443
Currently unmarried	0.71	0.5100 0.9990	0.049
High socioeconomic class	1.50	0.8034 - 2.8629	0.190
High educational level	2.55	1.0237 - 6.375	0.045
Resides in urban/semi urban areas	1.38	1.0174 - 1.8751	0.039^*^
Cigarette smoking	0.97	0.7297 - 1.2857	0.819
Drank alcohol	0.84	0 .6594 - 1.0915	0.192
BMI ≥ 25kg/m^2^	3.72	1.4744 - 9.4004	0.007^*^
Absence of diabetes mellitus	1.10	0.5701 - 1.9882	0.389

BMI- Body Mass Index, CI - Confidence Interval

* Statistically significant

**Table 3:** Demographic and clinical characteristics of subjects based on awareness of their hypertension status

Parameters	Hypertensive (Aware) N = 214	Hypertensive (Not Aware) N = 759	P-Value
**Gender**			0.560
Female	118(55.1%)	454(59.8%)	
Male	96(44.9%)	305(40.2%)	
**Age Group (years)**			0.003
65 - 69	68 (31.8%)	136 (17.9%)	
70 - 74	65 (30.4%)	211 (27.8%)	
75 - 79	34 (15.9%)	132 (17.4%)	
80+	47 (21.9%)	280 (36.9%)	
**Marital status**			0.260
Currently married	126 (58.9%)	377 (49.7%)	
Not currently married	88 (41.1%)	382 (50.3%)	
**Educational level**			0.145
No formal education	85 (39.7%)	357 (47.0%)	
Below secondary level	110 (51.4%)	356 (46.9%)	
Above secondary level	19 (8.9%)	46 (6.1%)	
**Economic class**			0.001^*^
< 3	85 (9.8%)	357 (25.3%)	
3-5	48 (34.8%)	143 (40.5%)	
6-10	24 (38.6%)	75 (28.4%)	
>10	19 (16.8%)	46 (5.8%)	
**Site of study**			0.107
Urban/ Semi Urban	170 (79.5%)	549 (72.3%)	
Rural	44 (21.5%)	210 (29.7%)	
**Ever smoked cigarette**			0.184
Yes	75 (35.0%)	306 (40.3%)	
No	139 (65.0%)	453 (59.7%)	
**Ever drank alcohol**			0.940
Yes	93 (43.5%)	285 (37.6%)	
No	121 (56.5%)	474(62.4%)	
**Body Mass Index (kg/m^2^)**			0.001^*^
< 18.5	21 (9.8%)	120 (15.8%)	
18.5 - 24.9	94 (43.9%)	446 (58.8%)	
25 - 29.9	61 (32.6%)	140 (18.4%)	
> 30	38 (17.7%)	53 (47.0%)	
**Diabetes mellitus**			0.007^*^
Present	20 (9.3%)	23 (3.0%)	
Absent	194 (90.7%)	736 (97.0%)	
**Transient ischaemic attack**			0.414
Previous TIA	15 (7.0%)	31(4.1%)	
No TIA	199 (93.0%)	728 (95.9%)	
**Dementia**			0.049^*^
Present	10(4.7%)	73 (9.6%)	
Absent	204 (95.3%)	686 (90.4%)	
**Depression in last 12 months**			0.985
Present	18(9.8%)	80(9.8%)	
Absent	166(90.2%)	734(90.2%)	

TIA - Transient Ischaemic Attack.

* Statistically significant.

In multivariate analysis, being in low socioeconomic class (OR 1.1, 95% CI 1.03 - 1.08, P < 0.001), having BMI ≥ 25kg/m^2^ (OR 3.01, 95% CI 1.36 - 7.09, P < 0.001) and not reporting the presence of diabetes mellitus (OR 3.01, 95% CI 1.30 - 7.12, P < 0.012), independently increased the likelihood of not being aware of the presence of hypertension (Table 4). Among those who were aware of and on treatment for hypertension, 165 (77.1%) were uncontrolled. In univariate analysis BMI ≥ 25kg/m^2^ and the experience of major depression in the previous 12 months were associated with poor blood pressure control (Table 5). In multivariate analysis BMI ≥ 25kg/m^2^ (OR 3.11, 95% CI 1.36 - 7.02) and low socioeconomic class (OR 8.21, 95% CI 1.56 - 9.15) independently increased the likelihood of having poor blood pressure control (Table 6).

**Table 4:** Factors independently associated with awareness of hypertension

Factors	Odd Ration	95% CI	P-value
Female gender	1.19	0.7403 - 1.9044	0.463
Age > 69 years	1.10	0.6981 - 1.6862	0.708
Currently unmarried	1.05	0.5950 - 1.9767	0.784
High socioeconomic class	8.21	3.7194 - 18.1088	0.001^*^
Resides in rural areas	1.34	0.8735 - 2.0662	0.171
Cigarette smoking	0.73	0.4487 - 1.1904	0.199
Drank alcohol	1.42	0.9191 - 2.1817	0.351
BMI ≥ 25kg/m^2^	3.11	1.3615 - 7.0923	0.009^*^
Absence of diabetes mellitus	3.01	1.3032 - 7.1145	0.012^*^

BMI- Body Mass Index, CI – Confidence Interval

* Statistically significant

**Table 5:** Comparative clinical characteristics of patients who were aware of their hypertension status, stratified according to the blood pressure control

Parameters	Hypertensive (Aware but not Controlled) N = 165	Hypertensive (Aware and controlled) N = 49	P-Value
**Gender**			0.270
Female	95 (57.6%)	23 (46.9%)	
Male	70 (42.4%)	26 (53.1%)	
**Age Group (years)**			0.815
65 - 69	52 (31.5%)	16 (32.7%)	
70 - 74	48 (29.1%)	17 (34.7%)	
75 - 79	23 (13.9%)	11 (22.4%)	
80+	42 (25.5%)	5 (10.2%)	
**Marital status**			0.329
Currently married	93 (56.4%)	16 (29.6%)	
Currently not married	72 (43.6%)	33 (67.4%)	
**Educational level**			0.480
No formal education	71 (43.0%)	14 (40.7%)	
Below secondary level	81 (49.9%)	20 (38.9%)	
Above secondary level	13 (7.9%)	6 (20.4%)	
**Economic Group**			0.353
< 3	16(9.7%)	8 (16.3%)	
3 -5	58(35.2%)	14 (28.6%)	
6 - 10	63(38.2%)	15 (30.6%)	
> 10	28(16.9%)	12 (24.5%)	
**Site of study**			0.939
Urban/ Semi Urban	133 (80.1%)	37 (74.1%)	
Rural	32 (19.9%)	12 (25.9%)	
**Ever smoked cigarette**			0.921
Yes	58 (35.1%)	17 (34.7%)	
No	108 (64.9%)	32 (65.3%)	
**Ever drank alcohol**			0.175
Yes	65(39.4%)	28 (57.1%)	
No	98(60.6%)	21 (42.9%)	
**Body Mass Index (kg/m^2^ )**			0.028^*^
< 18.5	15(9.1%)	6 (12.2%)	
18.5 - 24.9	69(41.8%)	25 (51.0%)	
25 - 29.9	52(31.5%)	8 (16.3%)	
> 30	24(14.5%)	4 (8.2%)	
**Diabetes mellitus**			0.458
Present	16(9.7%)	4 (8.2%)	
Absent	149(90.3%)	45 (91.8%)	
**Transient ischaemic attack**			0.592
Previous TIA	11(6.0%)	4 (11.1%)	
No TIA	173(94.0%)	45 (88.9%)	
**Dementia**			0.135
Present	8(4.8%)	2 (4.1%)	
Absent	157(95.2%)	47 (95.9%)	
**Depression in last 12 months**			0.002^*^
Present	18(9.9%)	21 (42.9%)	
Absent	147(89.1%)	28 (57.1%)	

TIA - Transient Ischaemic Attack.

* Statistically significant

**Table 6:** Factors independently associated with of hypertension control

Factors	Odd Ratio	95% CI	P-value
Female gender	0.72	0.3020 - 1.6997	0.423
Age > 69 years	1.28	0.2587 - 6.3060	0.749
Currently unmarried	0.78	0. 2904 - 1180	0.610
Low socioeconomic class	2.66	0.8706 - 8.1028	0.082
Resides in rural areas	1.10	0.5025 - 2.2736	0.852
Cigarette smoking	1.13	0.4166 - 3.0899	0.792
Drank alcohol	0.62	0.2351 - 1.6096	0.300
BMI ≥ 25kg/m^2^	5.51	1.4712 - 20.6536	0.015^*^
Diabetes mellitus	0.78	0.2229 - 2.7613	0.687
Depression (last 12 months)	0.81	0.3998 - 1.6538	0.544

BMI- Body Mass Index, CI - Confidence Interval

* Statistically significant.

## Discussion

The result of this study showed that hypertension is highly prevalent among elderly Nigerians and that the majority of affected individuals are females. Approximately 78% of persons with hypertension were not aware of the condition while among the few who were aware, the hypertension was uncontrolled in 77.1% despite reporting that they were on treatment. Factors independently associated with lack of awareness of hypertension included age less than 69 years, individuals with no self-reported history of diabetes mellitus and BMI ≥ 25kg/m^2^. Majority of the patients who were previously aware of their hypertension had poor blood pressure control. Having depression in the last 12 months and a BMI ≥ 25kg/m^2^ were independently associated with poor blood pressure control. The prevalence of hypertension in this population is much higher than in the general population, however the finding is similar to those of other studies among elderly populations [3]. The high prevalence of hypertension among elderly women when compared to men can be explained by the loss of the estrogen cardiovascular protecting effects after menopause. The high prevalence of hypertension has policy implications towards planning appropriate intervention for the growing health needs of the rising elderly population in the country. Hypertension was high among the urban and semi urban dwellers in this population and this is in agreement with reports from other previous studies [11–13]. The reasons for the high prevalence of hypertension in the urban settings could be attributed to constant exposure to stress, lack of or inadequate exercise and consumption of unhealthy diets, such as fast foods, which are high in salt and fat. These lifestyles are commoner among the urban population [21, 22]. The rate of awareness of hypertension in this population is similar to reports of awareness of hypertension among similar sub-Saharan African populations [23–26], but lower when compared to reported rates in studies from high income countries [27, 28]. The low awareness in this sample and in other previous reports from sub-Saharan Africa may be due to the low level of education among the studied populations. In the present sample, more than 60% of the population had no formal education or had educational level not higher than primary school. In addition, most of the participants resided in rural and semi-urban settings where access to health information and facilities are commonly limited [21, 29]. Individuals with self-reported diabetes mellitus were more like to be aware of their hypertension. Such persons would have had contacts with health facilities where thorough examination would have led to the hypertension being detected on time.

Individuals in the low socioeconomic class were less likely to be aware of their hypertension in this study and the relationship between hypertension awareness and socioeconomic status has been well documented by several studies [14, 28, 29]. The prevalence of hypertension and its awareness was higher in urban sub - population of the study compared to the rural population. This finding is similar to report from other studies [24, 29]. Among subjects with hypertension who were on treatment, only 22.7% were controlled. This is at variance with reports from high income countries [9–13, 27, 29]. The finding in our study is similar to the report by Akinkugbe et al who observed that only a third of persons with hypertension and on medications had optimal blood pressure control [30]. The low rate of hypertension control may be due to inadequate health education of the patients with hypertension, resulting from low doctor - patient ratio, busy clinic with attendant pressure on the time for consultation and counseling. Other factors that may contribute to poor BP control are the high cost of antihypertensive medications which might make them unaffordable in a setting where out-of-pocket payment for treatments is the rule. The large number of uncontrolled cases of hypertension suggests that many in the population have elevated risk of cardiovascular morbidity and mortality. This observation emphasizes the need for appropriate policies to be designed and implemented with a view to ensuring that hypertension is detected early and controlled through lifestyle modifications, optimal pharmacological treatments and assertive monitoring of adherence to treatments. Adoption of these measures among the elderly population has been shown to reduce morbidity and mortality associated with uncontrolled hypertension [31, 32].

## Conclusion

Our study shows high prevalence of hypertension among elderly Nigerians, majority of whom were not aware of the condition and high proportion of poor blood pressure control among those with previous diagnosis of hypertension. These findings emphasize the need for a re-evaluation of the current healthcare system in the country with a view to accommodating the health needs of the growing elderly population. The current advocacy for universal health coverage (UHC) which promises accessible and effective health care for all needs to be intensified in Nigeria in order to ensure that health policy makers in the country make adequate provisions for the needs of the elderly population. Our findings highlight the imperative of effective health education and treatment adherence monitoring strategies as important components of UHC.

### What is known about this topic

- There has been a steadily rise in elderly population in sub-Saharan Africa;
- There is high burden hypertension in the sub-Saharan African population;
- The burden of hypertension is highest among the elderly population.

### What this study adds

- Highlight the level awareness of hypertension among the elderly population which has not been previously well documented among the elderly sub-Saharan African population;
- Determine the prevalence of hypertension among the elderly population and compared it with other groups in the population;
- Identify comorbidities and factors associated with high burden of hypertension among the elderly population.

## Competing interests

The authors declare no competing interest.
